# High prevalence of multidrug resistant uropathogens: A recent audit of antimicrobial susceptibility testing from a tertiary care hospital in Bangladesh

**DOI:** 10.12669/pjms.36.6.2943

**Published:** 2020

**Authors:** Chandrika Dasgupta, Md. Abdur Rafi, Md. Abdus Salam

**Affiliations:** 1 Chandrika Dasgupta, MBBS. Intern Doctor, Shaheed Ziaur Rahman Medical College Hospital, Bogura, Bangladesh. Shaheed Ziaur Rahman Medical College, Bogura, Bangladesh; 2 Md. Abdur Rafi, MBBS. Intern Doctor, Rajshahi Medical College Hospital, Rajshahi, Bangladesh; 3 Md. Abdus Salam, PhD, FRCP (UK). Department of Microbiology, Present Address: Department of Basic Medical Sciences, Kulliyyah (Faculty) of Medicine, International Islamic University Malaysia, 25200 Kuantan, Pahang, Malaysia. Shaheed Ziaur Rahman Medical College, Bogura, Bangladesh

**Keywords:** Antibiogram, Multidrug resistant uropathogens, Tertiary care hospital, Bangladesh

## Abstract

**Objectives:**

Urinary tract infections due to multi drug resistant bacteria have been on the rise globally with serious implications for public health. The objective of this study was to explore the prevalence of multi drug resistant uropathogens and to correlate the urinary tract infections with some demographic and clinical characteristics of patients admitted in a tertiary care hospital in Bangladesh.

**Methods:**

A cross sectional prospective study was conducted at Shaheed Ziaur Rahman Medical College Hospital, Bogura, Bangladesh among clinically suspected urinary tract infection patients from January to December, 2018. Clean-catch midstream or catheter-catch urine samples were subjected to bacteriological culture using chromogenic agar media. Antimicrobial susceptibility testing of the isolates was done by Kirby-Bauer disk diffusion method following Clinical and Laboratory Standards Institute guidelines. Descriptive statistical methods were used for data analysis.

**Results:**

Culture yielded a total of 537 (42.8%) significant bacterial growths including 420 (78.2%) multi drug resistant uropathogens from 1255 urine samples. *Escherichia coli* was the most common isolate (61.6%) followed by *Klebsiella spp*. (22.5%), *Pseudomonas spp*. (7.8%), *Staphylococcus aureus* (5.4%) and *Enterobacter spp*. (2.6%) with multi drug resistance frequency of 77.6%, 71.9%, 90.5%, 86.2% and 92.9% respectively. There was female preponderance (M:F; 1:1.97; P=0.007) but insignificant differences between paediatric and adult population (43.65% vs. 42.57%) and also among different age groups. Diabetes, chronic renal failure, fever and supra-pubic pain had significant association as co-morbidities and presentations of urinary tract infections (P<0.05). Multi drug resistance ranged from 3.7 to 88.1% including moderate to high resistance found against commonly used antibiotics like ciprofloxacin, cephalosporin, azithromycin, aztreonam, cotrimoxazole and nalidixic acid (28.6 to 92.9%). Isolates showed 2.4 to 32.2% resistance to nitrofurantoin, amikacin, netilmicin and carbapenems except *Pseudomonas spp*. (66.7% resistance to nitrofurantoin) and *Enterobacter spp*. (28.6 to 42.9% resistance to carbapenems).

**Conclusion:**

There is very high prevalence of multi drug resistant uropathogens among hospitalized patients and emergence of carbapenem resistance is an alarming situation. Antibiotic stewardship program is highly recommended for hospitals to combat antimicrobial resistance.

## INTRODUCTION

Urinary tract infection (UTI) is among the most frequent bacterial infections in clinical practice worldwide. The frequency and burden of UTI is mostly underestimated and speculated to be higher than available data because it is not among mandatory notifiable diseases.^1^ A spectrum of clinical scenario from asymptomatic bacteriuria to complicated infections are observed in UTI affecting different age groups. Dysuria with or without frequency, urgency, and suprapubic pain are the usual accompaniments of lower urinary tract infections while complicated UTI like pyelonephritis usually presents with systemic symptoms like fever, chill, flank pain, hematuria and delirium.^2^ *Escherichia coli* remains the predominant uropathogen (80%) followed by *Klebsiella*, *Enterobacter*, *Proteus*, *Pseudomonas* and *Enterococci*. The pathogens traditionally associated with UTI are on a change particularly because of growing antimicrobial resistance and underlying host factors.^3^

UTI is becoming increasingly difficult to treat owing to high recurrence and multi drug resistant (MDR) uropathogens especially extended-spectrum β-lactamases (ESBLs) producing bacteria that have been on the rise globally with serious implications for public health.^4^ Traditionally broad spectrum antibiotics remain the drug of choice to treat UTI but indiscriminate use of antibiotics is making the treatment challenging as it accelerates the emergence of drug resistant bacteria. This practice is more common in lower and middle income countries like Bangladesh, where empirical therapy is a common practice as the laboratory facility for urine culture is not widely available.^5^ Although bacterial etiology of UTI and antimicrobial resistance pattern may have regional variations and largely depend on the antibiotic policy of health care facility but the growing frequency of MDR uropathogens has become now a universal problem and more alarming in the developing countries including Bangladesh.^6^,^7^

Routine antimicrobial susceptibility testing is a pre-requisite not only to choose the appropriate antibiotic but also to facilitate the empiric therapy.^8^ The present investigation was carried out to explore the current prevalence of MDR uropathogens, frequency of resistance to different classes of antibiotics and to correlate UTI with patient’s demographic and clinical characteristics from a tertiary care hospital in Bangladesh.

## METHODS

This cross sectional prospective investigation was conducted from January to December, 2018 among one thousand two hundred fifty-five (1255) admitted patients of different age and gender of Shaheed Ziaur Rahman Medical College Hospital (SZMCH), Bogura, a 1000-bed tertiary care teaching hospital in the Northern part of Bangladesh. Patients with at least one of the clinical features of UTI (fever, dysuria, frequency, urgency, supra-pubic pain, loin pain, haematuria, nocturia or prior history of UTI) were judged as clinically suspected and selected for confirmation by laboratory tests. Clean-catch midstream or catheter-catch urine was collected into a sterile wide mouth container/test tube with all aseptic measures and was screened for microscopic demonstration of pus cells ≥ 5/ HPF (high power field) in a centrifuged deposit before considering culture.^9^

Ethical Review Committee of Shaheed Ziaur Rahman Medical College, Bogura, Bangladesh approved the protocol and informed written consent/assent was taken from patient (Ref: SMZC/2016/351, Dated: 10-12-2016).

### Urine culture

Chromogenic agar (HiMedia, India) medium was used for urine culture and samples were inoculated aseptically using a calibrated wire loop of 28G (internal diameter 3.26 mm) holding 0.004 ml of urine for overnight aerobic incubation at 37^0^C. Details of culture technique, significant bacteriuria and identification methods have been reported previously.^10^ The final identification of the isolates was done using standard identification protocol such as Gram’s staining, motility test, catalase test, coagulase test, oxidase test and relevant biochemical tests as appropriate for the isolates.^11^ Diagnosis of UTI was established on the basis of presenting feature(s), pyuria and significant bacteriuria.

### Antimicrobial susceptibility testing (AST

ueller-Hinton agar and Kirby-Bauer disk diffusion method^12^ were used for AST against a panel of 17 commercial antibiotic disks (Oxoid, UK): amikacin (30µg), azithromycin (15µg), aztreonam (30μg), cefuroxime sodium (30µg), cefixime (5µg), cefepime (30µg), ceftazidime (30µg), ceftriaxone (30µg), ciprofloxacin (10μg), cotrimoxazole (25µg), gentamicin (30μg), imipenem (10µg), levofloxacin (5μg), meropenem (10µg), nalidixic acid (30µg), netilmicin (30μg) and nitrofurantoin (300µg). Isolates were labeled as ‘sensitive’ and ‘resistant’ according to the guidelines of Clinical Laboratory Standard Institute (CLSI). *Escherichia coli* ATCC 25922 and *Staphylococcus aureus* ATCC 25923 were used as control strains for AST.^13^ Resistance to at least one agent in three or more antimicrobial categories was defined as multi drug resistance.^14^

### Data collection and statistical analysis

A structured questionnaire was used for patient’s demographic and clinical data. Descriptive statistical methods in SPSS (version 21.0 for Windows, SPSS® Inc., Chicago, IL) were applied for data analysis. The antimicrobial resistance prevalence was calculated as the proportion of positive results against total sample. Nominal variables were shown as number of cases (*n*) and percentage (%). Test of significance was performed using the Chi-square (χ^2^) test and variables including demographic and clinical characteristics of UTI patients were compared using cross-tabulation statistical methods. *P* < 0.05 was considered statistically significant.

## RESULTS

Culture yielded 537 (42.8%) as positive out of 1255 clinically suspected UTI patients. *Escherichia coli* was the most common (61.6%) gram negative isolate followed by *Klebsiella spp*. (22.5%), *Pseudomonas spp*. (7.8%) and *Enterobacter spp*. (2.6%), while *Staphylococcus aureus* (5.4%) was the only gram positive isolate. Of 537 isolates, 420 (78.2%) were found to be MDR with frequency distribution of *Escherichia coli*, *Klebsiella spp*., *Pseudomonas spp., Staphylococcus aureus* and *Enterobacter spp*. was 77.6%, 71.9%, 90.5%, 86.2% and 92.9% respectively (Table-I).

**Table-I:** Frequency of culture-positive (n = 537) and MDR (n = 420) uropathogens.

Uropathogens	Frequency of culture-positive isolates n (%)	Frequency of MDR isolates n (%)
Escherichia coli	331 (61.6)	257 (77.6)
Klebsiella spp.	121 (22.5)	87 (71.9)
Pseudomonas spp.	42 (7.8)	38 (90.5)
Staphylococcus aureus	29 (5.4)	25 (86.2)
Enterobacter spp.	14 (2.6)	13 (92.9)

Total	537 (100)	420 (78.2)

There was female preponderance (M:F; 1:1.97) among culture-positive cases and statistically highly significant (P=0.007). Age distribution revealed 252 (20%) patients from paediatric age group (up to 18 yrs.) and 1003 (80%) adults with no significant difference in frequency of culture-positive cases (43.7% vs. 42.6%). Also there was no statistical significance in rate of isolation among different age groups. Co-morbid conditions like hypertension (19.8%), diabetes mellitus (18.4%), ischaemic heart disease (4.1%), chronic obstructive pulmonary disease (3.9%), chronic renal failure (3.1%) and asthma (2.2%) were noted among UTI patients with significant (P < 0.05) association observed for diabetic and chronic renal failure patients. Among symptoms, dysuria (79%), frequency (53.5%), fever (33.7%), urgency (31.7%), supra-pubic pain (26.5%), nocturia (14.5%) and haematuria (4.9%) were noted with fever and supra-pubic pain had significant (P < 0.05) association with UTI (Table-II).

**Table-II:** Correlation of UTI with demographic and clinical characteristics of the patients.

Characteristics	Culture-positive n(%)	Culture-negative n(%)	Total n(%)	P-value
***Gender***				
Female	356 (66.3)	422 (58.8)	778 (62.0)	0.007
Male	181 (33.7)	296 (41.2)	477 (38.0)	
***Age***				
< 5 years	70 (13.0)	92 (12.8)	162 (12.9)	0.994
5 – 18 years	40 (7.4)	50 (7.0)	90 (7.2)	
19 – 30 years	95 (17.7)	135 (18.8)	230 (18.3)	
31 – 40 years	65 (12.1)	92 (12.8)	157 (12.5)	
41 – 50 years	71 (13.2)	98 (13.6)	169 (13.5)	
51 – 60 years	83 (15.5)	104 (14.5)	187 (14.9)	
> 60 years	113 (21.0)	147 (20.5)	260 (20.7)	
***Chronic Medical Conditions***				
Hypertension	108 (20.1)	140 (19.5)	248 (19.8)	0.487
Diabetes mellitus	139 (25.8)	92 (12.8)	231 (18.4)	0.004
Ischaemic heart disease	20 (3.7)	32 (4.4)	52 (4.1)	0.364
Chronic obstructive pulmonary disease	21 (3.9)	28 (3.8)	49 (3.9)	0.148
Chronic renal failure	23 (4.2)	16 (2.2)	39 (3.1)	0.029
Asthma	11 (2.1)	17 (2.3)	28 (2.2)	0.774
***Clinical presentation***				
Dysuria	432 (80.5)	559 (77.8)	991 (79.0)	0.372
Frequency	296 (55.1)	375 (52.2)	671 (53.5)	0.143
Fever	257 (47.8)	166 (23.2)	423 (33.7)	0.012
Urgency	192 (35.7)	206 (28.7)	398 (31.7)	0.271
Supra-pubic pain	158 (29.4)	174 (24.2)	332 (26.5)	0.048
Nocturia	84 (15.6)	98 (13.6)	182 (14.5)	0.153
Haematuria	30 (5.6)	32 (4.4)	62 (4.9)	0.076

MDR ranged from 3.7 to 88.1% and isolates were found to be moderate to highly resistant (28.6 to 92.9%) to commonly used antibiotics like ciprofloxacin, cotrimoxazole, azithromycin, nalidixic acid, cephalosporin and aztreonam (Table-III). Nitrofurantoin, amikacin, netilmicin, imipenem and meropenem were found to be better choice (resistance ranged from 2.4 to 32.2%) for all isolates, except *Pseudomonas spp*. and *Enterobacter spp*. Nitrofurantoin resistance was 66.7% for *Pseudomonas spp*. while *Enterobacter spp*. showed moderate resistance to both imipenem (28.6%) and meropenem (42.9%).

**Table-III:** Frequency of antimicrobial resistance of uropathogens (n = 537).

Antimicrobial Agents	Escherichia coli (n = 331)	Klebsiella spp. (n = 121)	Pseudomonas spp. (n = 42)	Staphylococcus aureus (n = 29)	Enterobacter spp. (n = 14)	Total MDR n (%)
Cefixime	187 (56.5)	74 (61.2)	25 (59.5)	12 (41.4)	11 (78.6)	309 (57.5)
Ceftriaxone	166 (50.2)	69 (57.0)	16 (38.1)	3 (10.3)	7 (50.0)	261 (48.6)
Ceftazidime	128 (38.7)	53 (43.8)	12 (28.6)	9 (31.0)	10 (71.4)	212 (39.5)
Cefepime	158 (47.7)	57 (47.1)	13 (31.0)	10 (34.5)	10 (71.4)	248 (46.2)
Amikacin	29 (8.8)	16 (13.2)	2 (4.8)	2 (6.9)	3 (21.4)	52 (9.7)
Gentamicin	134 (40.5)	55 (45.5)	22 (52.4)	13 (44.8)	9 (64.3)	233 (43.4)
Imipenem	9 (2.7)	5 (4.1)	1 (2.4)	1 (3.4)	4 (28.6)	20 (3.7)
Meropenem	8 (2.4)	9 (7.4)	2 (4.8)	2 (6.9)	6 (42.9)	27 (5.0)
Ciprofloxacin	189 (57.1)	46 (38.0)	14 (33.3)	18 (62.1)	10 (71.4)	277 (51.6)
Levofloxacin	173 (52.3)	41 (33.9)	12 (28.6)	4 (13.8)	10 (71.4)	240 (44.7)
Azithromycin	178 (53.8)	66 (54.5)	20 (47.6)	18 (62.1)	9 (64.3)	291 (54.2)
Cefuroxime	154 (46.5)	70 (57.9)	26 (61.9)	18 (62.1)	9 (64.3)	277 (51.6)
Aztreonam	131 (39.6)	61 (50.4)	12 (28.6)	24 (82.8)	13 (92.9)	241 (44.9)
Cotrimoxazole	192 (58.0)	66 (54.5)	35 (83.3)	20 (69.0)	8 (57.1)	321 (59.8)
Nitrofurantoin	68 (20.5)	39 (32.2)	28 (66.7)	5 (17.2)	3 (21.4)	143 (26.6)
Netilmicin	60 (18.1)	38 (31.4)	10 (23.8)	8 (27.6)	3 (21.4)	119 (22.2)
Nalidixic Acid	292 (88.2)	110 (90.9)	35 (83.3)	24 (82.8)	12 (85.7)	473 (88.1)

N.B. Figures in the parentheses indicate percentage.

## DISCUSSION

Resistance to commonly used antibiotics is an emerging concern worldwide causing treatment failure in different infections including UTI.^15^ The present study highlights a baseline evidence on current situation of very high frequency of MDR uropathogens among hospitalized patients. It is no wonder that we found female preponderance which is an established fact that due to many inherent host factors females are more vulnerable to UTI.^4^ Diabetes and chronic renal failure were found to have statistically significant association with UTI and these findings corroborate well with other reports.^16^,^17^ Predisposition to UTI in diabetes results from several factors including increased susceptibility to uncontrolled glycaemia and defective host immunity. While chronic renal failure is a risk factor for UTI due to metabolic disorders resulting in secondary immunodeficiency.

Fever and supra pubic pain had significant association among others as presentations of UTI. Fever usually denotes pyelonephritis and more commonly observed in paediatric patients while supra pubic pain indicates cystitis which is common among lower UTI.^18^ *Escherichia coli* was the most frequent isolate followed by *Klebsiella spp*., which is consistent with many previous studies.^4^,^6^,^8^,^10^ In fact, *E. coli* stands alone for around 70% as the etiological agent of both community and hospital acquired UTI.^4^ We found *Staph aureus* as only gram positive uropathogen and it coincides with other report.^19^ Although coagulase negative *Staph saprophyticus* has been reported as frequent uropathogen usually from patients attending outpatient or antenatal clinics but it contradicts with ours because we included hospital admitted cases where *Staph aureus* is a common pathogen.^4^,^5^,^17^

High prevalence of MDR uropathogens with moderate to high resistance to most of the common antimicrobial classes including cephalosporin, quinolone and fluoroquinolones, macrolide, cotrimoxazole and injectable drugs like aztreonam and gentamicin was observed in the present study which is higher in comparison to our previous report.^5^ This difference can be correlated with inclusion of hospital acquired UTI in the present setting where there is high frequency of MDR uropathogens.^20^ Comparable prevalence rate of MDR uropathogens to commonly prescribed antimicrobials has also been reported by some recent studies.^21^,^22^

We found nitrofurantoin and amikacin as better choice except *Pseudomonas spp*. which is consistent with our previous report and other recent studies.^5^,^23^,^24^ Reasons for the emergence of low resistance to nitrofurantoin are not fully understood, but likely include its restricting use, limited systemic absorption and the need for multiple genetic mutations in bacteria to develop resistance. The most important and alarming findings of the present study are variable degree of resistance to carbapenems ranging from 2.4 to 42.9% shown by different isolates which corroborates with others.^23^,^25^ Carbapenems are the latest developed β-lactam containing broad spectrum antibiotics usually reserved for MDR pathogens. Lately, the dissemination of community acquired *E. coli* capable of producing ESBLs that can hydrolyze almost all β-lactams except carbapenems has been reported worldwide; consequently, the use and abuse of carbapenems have increased greatly with emergence of carbapenem resistance as a serious concern. Evidence based choice of antibiotics from *in-vitro* antimicrobial susceptibility test may reduce the risk of this alarming situation when there is current shortage of effective therapies, lack of successful preventive measures and of course very slow development of novel treatment options. Further, practice of Antibiotic Stewardship Programs (ASPs) in hospitals can help minimizing the problem of antibiotic resistance and it has been recommended by the Centers for Disease Control and Prevention (CDC). ASPs refer to optimizing the use of antibiotics to effectively treat infections, protect patients from harms caused by unnecessary antibiotic use and combat antibiotic resistance. The programs have at least seven core components to help clinicians to improve clinical outcomes by improving antibiotic prescribing practice (https://www.cdc.gov/antibiotic-use/healthcare/pdfs/hospital-core-elements-H.pdf).

### Limitations of the study

First of all, it was a single center study, so the findings cannot be generalized in the national context. Second, the detailed clinical classification of UTI patients could not be mentioned. Third, the genotypic or phenotypic categorization of MDR uropathogens were not established.

## CONCLUSIONS

Increasing frequency of MDR uropathogens is a global concern and has been reinforced by our findings. Commonly prescribed antibiotics including third generation cephalosporin and fluoroquinolones have shown to be poorly efficacious while nitrofurantoin, netilmicin and amikacin still hold some promise. Although the reserve drugs like carbapenems are still the best choice in treating the hospital acquired UTI but emergence of carbapenem resistance is a great concern. Antibiotic Stewardship Programs (ASPs) can only optimize the use of antibiotics to effectively treat infections and to improve clinical outcomes. We strongly advocate the introduction of ASPs in the tertiary care hospitals in Bangladesh to combat antibiotic resistance.

### Author’s Contributions

**CD & MAS:** conceived, designed and editing of the manuscript.

**MAR & CD:** data collection, analysis and manuscript writing.

**MAS:** responsible for the accuracy and integrity of the work.

All authors have read and approved the final form of the manuscript.
